# Cholera Infantum

**Published:** 1877-07

**Authors:** 


### CHOLERA INFANTUM.

The very mention of this disease is a dread in every household fortunate enough to be blessed with a darling baby. This is the season of year when deaths from this cause, figure largely in our mortality reports, bringing anxiety to every parent, lest it may invade their own happy home. The disease is most prevalent in cities, and during the months of July and August, when the extreme heat has a depressing influence upon the young babe. This circumstance, coupled with the administration of improper food, as fruit or berries, or the accidental giving of sour milk, are common causes for the attack. Infants of two years, or under, are the usual victims of the affection, their little stomachs being very sensitive to any improperly prepared food. The hygienic condition of the premises upon which the infant abides is an important factor in the case. Parents cannot be too particular to see that the cellar under their dwelling, is entirely free from any dampness, or decomposing vegetable matter, and that it is thoroughly ventilated. Noxious gases, arising from cess-pools, or house-drains, or from rooms overcrowded, are conditions under which the disease thrives.

Too much precaution cannot be taken with reference to the cleanliness and sweetness of the bottle from which the infant is fed, as well also, as the cup in which its food is warmed or prepared. The least particle of sour milk, that might adhere to bottle or cup, would be sufficient to originate the disease. Avoid taking the infant in damp places.—as in a parlor shut from the light and air for several days, or in the night air. Its sleeping apartment should be the brightest, sunniest and largest in the house, and located in the second story, if possible. These be a few of the precautions that may be taken to prevent an attack of this dreadful disease, but when it *does* come, lose no time in summoning your family physician, for, upon his early arrival and management of the case, depends much of the success of its treatment. Always seek immediate advice when your infant has thin or watery stools, at this season of the year, accompained with vomiting or irritabitity of the stomach. Do not attribute these symptoms to "teething," regarding them therefore, as trivial, soon to pass away. They are always of an alarming character, demanding prompt measures for their arrest, ere the infant becomes too weak and exhausted to rally under any of the well directed efforts of the medical man. Act promptly.

